# Surveillance and Monitoring of Dialysis Access

**DOI:** 10.1155/2012/649735

**Published:** 2011-11-22

**Authors:** Lalathaksha Kumbar, Jariatul Karim, Anatole Besarab

**Affiliations:** Division of Nephrology and Hypertension, Henry Ford Hospital, Detroit, MI 48202, USA

## Abstract

Vascular access is the lifeline of a hemodialysis patient. Currently arteriovenous fistula and graft are considered the permanent options for vascular access. Monitoring and surveillance of vascular access are an integral part of the care of hemodialysis patient. Although different techniques and methods are available for identifying access dysfunction, the scientific evidence for the optimal methodology is lacking. A small number of randomized controlled trials have been performed evaluating different surveillance techniques. We performed a study of the recent literature published in the PUBMED, to review the scientific evidence on different methodologies currently being used for surveillance and monitoring and their impact on the care of the dialysis access. The limited randomized studies especially involving fistulae and small sample size of the published studies with conflicting results highlight the need for a larger multicentered randomized study with hard clinical end points to evaluate the optimal surveillance strategy for both fistula and graft.

## 1. Introduction

Vascular access is the lifeline of a hemodialysis patient. The evolution of vascular access has come a long way since the days of Scribner Shunt [1]. Currently arteriovenous fistula (AVF) and arteriovenous graft (AVG) have been recognized as the permanent accesses for a dialysis patient with tunneled cuffed catheter (TCC) being the bridge to obtain a permanent access. Fistula First Breakthrough Initiative with its efforts to highlight the importance of autologous arteriovenous fistula and to educate the nephrologists, vascular surgeons, and patients has yielded a progressive improvement in the number of patients who are currently using the fistula for hemodialysis. In May 2011, the national arteriovenous fistula rate reached 58.6% [2]. Though we have increased the use of the autologous arteriovenous fistula, a number of complications such as thrombosis, infection, stenosis, and access loss have plagued the care of these accesses. Vascular access failure has economic as well as adequacy of dialysis delivery implications. Measures taken for optimization of vascular access consumes about 8% of the Medicare spending on end-stage renal disease (ESRD), yet evidence on how to evaluate and treat the factors which affect the vascular access function is at best suboptimal [3]. Meanwhile, vascular access problems like low blood flow rates and loss of patency are frequently noted in dialysis units. These issues and other complications lead to extended treatment times, underdialysis, and frequent hospitalizations [4]. 

The Dialysis Outcome Quality Initiative Guidelines (DOQI) published by the National Kidney Foundation has provided a list of techniques which could be applied for monitoring and surveillance of vascular accesses [5]. Center for Medicare and Medicaid Services (CMS) mandates that both monitoring and surveillance be part of the dialysis care being provided to the ESRD patients with an aim of identifying and intervening at an early stage, with the intent of controlling the spiraling costs of access care [6]. Though various techniques are in use for this purpose, no clear consensus has been reached regarding the most optimal surveillance technique which identifies a failing access of all types. We performed a systematic literature review to identify various surveillance techniques and its effects on access function outcomes.

## 2. Methods and Results

In order to understand the available surveillance techniques and their effects on vascular access outcomes, we performed a PUBMED search through July 2011 of articles in English language, limited to the last 20 years, and available as full articles. The following MeSH terms used in the search “hemodialysis vascular access” [All Fields] OR “hemodialysis vascular access monitoring” [All Fields] OR “haemodialysis” [All Fields] OR “renal dialysis” [MeSH Terms] OR “renal” [All Fields] AND “dialysis” [All Fields] OR “renal dialysis” [All Fields] OR “hemodialysis” [All Fields] AND “blood vessels” [MeSH Terms] OR “blood” [All Fields] AND “vessels” [All Fields] OR “blood vessels” [All Fields] OR “vascular” [All Fields] AND access [All Fields] AND “epidemiology” [Subheading] OR “epidemiology” [All Fields] OR “surveillance” [All Fields] OR “epidemiology” [MeSH Terms] OR “surveillance” [All Fields] OR transonic [All Fields] AND access [All Fields] AND flow [All Fields] OR differential [All Fields] AND conductivity [All Fields] AND technique [All Fields] OR clinical [All Fields] AND monitoring [All Fields] AND “haemodialysis” [All Fields] OR “renal dialysis” [MeSH Terms] OR “renal” [All Fields] AND “dialysis” [All Fields] OR “renal dialysis” [All Fields] OR “hemodialysis” [All Fields] AND access [All Fields]. This resulted in 4412 publications. We then identified, reviewed, and extracted those studies which evaluated the various surveillance techniques, either comparing different surveillance modalities or were randomized studies. We then focused on those studies in which access outcome was the primary objective. We found only 7 studies with randomization and 17 studies where a cohort of patients was used. All studies were prospective with access outcome as an end point. There were six studies which evaluated only the autologous AVF, eight studies about AVG, and 10 studies where AVF and AVG were combined in the primary analysis. The discussion below summarizes the findings and conclusions from these studies.

## 3. Discussion

### 3.1. Monitoring and Surveillance Techniques

Monitoring strategies include physical examination (inspection, palpation, and auscultation) of the vascular access to detect physical signs that suggest the presence of physical pathology [7]. It also includes review of routine laboratory studies regularly obtained in the dialysis unit, dialysis adequacy (urea reduction ratio or Kt/V), and difficulties in cannulation or achieving hemostasis after needle withdrawal, documented recirculation, and other clinical clues. Physical examination of the access by an experienced individual has high sensitivity and specificity [8–10]. Measurement of dynamic venous pressure (DVP) during dialysis is currently considered as a monitoring strategy rather than a surveillance tool. Most of the modern dialysis machine measures the dynamic venous pressure during treatment, but the utility of dynamic venous pressure at flows 150–200 mL/min in detecting stenosis or predicting access thrombosis is very limited [11]. DVP is crucially dependent on the needle gauge and the length of the metallic portion of the dialysis needle. In addition, the length and the thickness of the needle shaft vary among manufacturers. In most dialysis units revalidation of the measurement procedures are usually not done with change of needle type [11, 12]. 

Surveillance, on the other hand, mandates periodic evaluation of the Vascular Access by means of specifically designed tests that may involve special instrumentation, for which an abnormal test result suggests the presence of pathology. Surveillance tests require additional time and effort from staff and in some circumstances dedicated technicians or nurses to yield consistent results. Access flow measurement [5, 13–15], duplex Doppler ultrasound [16–18], and direct or derived static pressure [19, 20] are the frequently used surveillance tools studied in the literature, flow measurement being the most widely used technique.

Access flow is measured by inducing forced recirculation where the arterial and venous blood lines are reversed. A signal is engendered either by infusion of a substance (saline, glucose), change in ultrafiltration rate (change in hematocrit), or addition of sodium (change in conductance) in the venous return line [14]. Most flow measurements are done at blood pump flows of 200–300 mL/min to avoid the increasing difference between actual blood flow and the blood pump flow at higher prepump pressure. During the interval of measurement, effective dialysis is reduced.

Duplex ultrasound studies (DUSs) can provide an independent accurate measure of blood pump blood flow. DUS measurement can be made in a few minutes producing virtually no effect on Kt/V, but routine use of it may be limited by cost and operator skill. The delta hematocrit method can reduce the effective treatment time for up to 8–10 minutes, whereas the conductivity-based method can take up to 20 minutes or more [15, 21].

Static venous pressure is another well-established technique for detecting physiologically significant stenosis in AVG [19, 22] and is able to reduce graft thrombosis [22, 23]. Its usefulness in predicting thrombosis or access failure in AVF is currently unknown. After initial description of the technique by Besarab et al., measurement of the static intra-access pressure (Pia) has evolved over time. Original method required a pressure transducer between the venous return tubing and the venous needle and connected to a pressure monitor. As intra-access pressure is influenced by mean arterial pressure (MAP), Pia is normalized to MAP as a ratio Pia/MAP. Pia/MAP ratio of 0.5 has a sensitivity of 81% and specificity of 80% in detecting a stenosis >50% by diameter [24]. The same group evolved a computerized method using the dynamic pressure readings taken during any dialysis session and extracting from it the static pressure while factoring out the contributions of chair heights, blood pump flow, and hematocrit [25]. The evolved method achieves the same result in AVF and AVG [19]. 

The rationale for monitoring and surveillance should be to improve longevity of the vascular access, reduce thrombosis rate and the use of temporary catheters. Understanding the pathophysiological effect of the stenosis is important in interpreting findings of monitoring and surveillance tools. Access dysfunction occurs mostly due to underlying stenosis. Stenosis eventually reduces access flow and alters the pressure profiles and is nearly always a prerequisite for access thrombosis [26, 27]. In reality access flow and pressures vary during and between dialysis sessions. Variation occurs due to cannulation technique, changes in hemodynamic among the dialysis sessions [28–30]. Therefore, a single measurement of either flows or pressure is not helpful in detecting an evolving stenosis [28]; rather multiple repetitive measurements are required [31–33]. The relationship between blood flow and intra-access pressure in a stenotic access depends on the location of the lesions [34]. One single technique may not be able to detect lesions at various locations that can occur in an access. Frequently multiple lesions are common in the territory of a vascular access, and the physiologic effect produced will depend on whether these are simple lesion at the inflow or outflow of the access or mixed (both inflow and outflow), their time of occurrence, and the progression of the stenosis independently over time or concurrently [31, 35]. In general an outflow stenosis causes an increase in intra-access pressure and overtime decreases access flow [36]. Clinically it can be manifested as prolonged postneedle withdrawal bleeding, aneurismal dilatation, and development of recirculation. This is particularly more evident in AVG than in AVF. In AVF some of the intra-access pressure can be dissipated by the development of collaterals. Determination of the rate of progression of the stenotic lesions is crucial for timing of intervention and to prevent unnecessary intervention. Angioplasty of the subclinical stenosis does not improve access outcome rather could promote stenosis [37]. Therefore, sequential measurement of pressure or flow or both is required to identify accesses at risk which will need intervention. The effect of inflow stenosis differs from outflow lesions. With inflow stenosis intra-access pressure either remains stable or decreases and the access flow may decrease without any change in the prepump pressure setting of the dialysis machine [36]. Surveillance tools based on pressure monitoring may not be able to detect such stenosis. But it can be detected by sequential flow measurement or physical examination [31, 32]. 

 The study conducted by Tessitore et al. [34] indicates that the best test to detect a given stenosis depends on its location. Flow measurement is useful for identifying inflow stenosis, whereas derived static venous pressure is a better tool for outflow lesions. As mentioned before, an access can have multiple lesions involving both inflow and outflow. It is, therefore, imperative to implement a process rather than a single method in detecting stenosis.

Vascular accesses are abandoned in large part due to irreversible thrombosis which in many times is preceded by one or more episodes of reversible thrombosis. This is especially true for AVG. In several observational studies, it was noted that the primary patency of the graft after elective angioplasty (70% to 85%) is superior to angioplasty after thrombectomy (37% to 63%) [38]. This finding favors implementation of a surveillance method to detect graft stenosis prior to thrombosis and preemptive angioplasty to improve graft survival. In search of an optimal surveillance tool, many observational studies have been conducted comparing different surveillance techniques and their ability to identify accesses at risk.

We should keep in mind that an abnormal surveillance data should always be correlated with clinical findings to determine the need for referral for intervention. At present there is little quality assurance for the success of intervention other than anatomical success. At most access center, peri-procedural assessment of intra-access pressure or flow measurements are unavailable to be correlated with prediction of secondary access patency. Several studies, Tessitore et al. [39], Murray et al. [40], and Van der Linden et al. [41], found that higher post intervention Qa was the only variable associated with improved access longevity. Although both DOQI guideline and CMS mandate implementation of surveillance methods, they do not prefer one surveillance technique over another due to lack of sufficient evidence in the literature [5].

### 3.2. Observational Studies

#### 3.2.1. Intervention before Thrombosis through Surveillance

Four observational studies by May et al. [42], Wang et al. [43], Paulson et al. [44], and McDougal and Agarwal [45] tested the positive predictive value and sensitivity of the access flow in predicting graft thrombosis. In these studies only 25% to 43% of the grafts with baseline flow of <500 to 700 mL/min developed thrombosis over the next 3 months. Neyra et al. tested this hypothesis in a prospective manner. Their study showed only 26% of the AVG with a 25% decrease in access flow thrombosed over the next 3 months [46]. The accuracy of the correlations may be strongly influenced by the accuracy and timing of the access flow measurement. Flow measurements are time dependent and vary during dialysis as well as within dialysis sessions. The study conducted by Polkinghorne et al. [47] measured blood flow multiple times during the dialysis session for 3 consecutive sessions. They noted significant reduction in flow and MAP throughout the dialysis treatment in a progressive manner. Flow can decrease by 10–30.6% during the last hour of dialysis. Similar results were found by Huisman et al. [48] using duplex Doppler ultrasound and Doppler ultrasound studies methods. 

Besarab et al. [22] conducted a prospective observational study to test the utility of static venous pressure to detect and correct venous outlet stenosis prior to thrombosis. Observation period was quite long for 7.75 years, and a total of 832 patient-access years of risk was monitored. 65% to 80% of the accesses were prosthetic graft. The result of this study was very promising; static venous pressure/systolic BP was found to provide excellent criteria for angiographic referral and intervention of >50% stenosis using angioplasty or surgical revision. There was marked reduction of the thrombosis rate (70%) and access replacement rate (79%) compared with the historical baseline. Similar observational studies using different surveillance tools also showed promising results. Specifically Sands et al. [49] showed a 6.5-fold reduction in thrombosis rate from 1.25 to 0.19 events per patient year at risk (duplex ultrasound imaging) and Mccarley et al. [50] a 4.4-fold reduction from 0.71 to 0.16 (access flow). Both Hoeben et al. [51] and Glazer et al. [52] achieved a 2-fold reduction in thrombosis events, from 0.32 to 0.17- (using flow methodology).

 The utility of combining flow monitoring and static venous pressure was tested by another observational study conducted by Smits et al. [11]; this study fails to show any advantage of combining the 2 surveillance strategies. On the contrary, recent observational study by Plantinga et al. [53] on 363 prospectively followed incident dialysis patients did not find any advantage of using such surveillance. A similar finding was also observed by Shahin et al. [54]. 

In the era of automation, Zasuwa et al. have described a novel methodology using an automated noninvasive surveillance algorithm which incorporates the vascular access pressure ratios. They studied the thrombosis rates during a baseline 6-month period to the subsequent 6-month periods when the algorithm was applied. A vascular access pressure ratio of >0.55 was considered significant. No special instruments or clinical staff was required for this automated process which generated a warning list of patients who had abnormal results. After 18 months of implementation, the thrombosis rate decreased from 0.29 to 0.13 events per patient-access-year, an impressive 57% decrease [55]. 

### 3.3. Randomized Controlled Trials

Randomized controlled trials are the gold standard for evidence in medicine. Interventional nephrology is a relatively new subspecialty. Very few RCTs have been conducted involving the vascular access. Twelve RCTs have been published; eight of them describing outcomes in AVG and 4 in AVF. There are two additional studies on reanalysis of the published data. Nine studies compared surveillance and intervention versus usual clinical monitoring and intervention in 1363 participants [49, 53, 56–62], including two studies which were prospective cohort studies [53, 61]. Sample size of the individual trials ranged from 51 to 189 with a mean of 151 and a mean duration of 17 months (range of 6–28 months). The other five were trials of patients with abnormal surveillance results who are randomly allocated to intervention (either percutaneous or surgical) or usual clinical monitoring. These 5 trials included 336 participants with a follow-up period of 12–15 months [37, 63–66]. All of the studies have their own limitations concerning sample size, population characteristics, method of surveillance, poor reporting of allocation concealment, blinding, vintage of the access in use, recruitment criteria, and the method of intervention. See Table 1. 

Sands et al. [49] studied 103 patients (68 AVF and 35 AVG) in a randomized controlled study to see whether frequent monitoring on a monthly basis rather than 6 monthly evaluations minimize access thrombosis. They also compared the efficacy of the two surveillance techniques, access flow, and static venous pressure. The study populations were randomized into three groups: monthly measurement of access flow (Qa), monthly measurement of static venous pressure (VPS), or no monthly monitoring (control group). Color flow Doppler ultrasound was performed in all patients every 6 months. In the flow group criteria for referral were access flow <800 mL/min in AVG and <600 mL in AVF or a ≥25% decline in flow. In the static pressure monitoring group, static venous pressure ratios >0.5 were referred for angiography and angioplasty of >50% stenosis. Mean follow-up time was 197 days. Their study showed that intervention based on monthly surveillance decreased access thrombosis both in AVF and AVG (*P* < 0.01) compared to no monitoring. In this study, measurement of access flow tends to result in lower thrombosis rates than the static venous pressure. This study has several limitations. In regards to static venous pressure, they used the same intervention criteria for fistula and graft, as we know that fistulae have lower static venous pressure than AVG and remain patent at a low flow state [67]. Moreover, the criteria for intervention were based upon changes in flow rate (≥25% decline in flow rate) but not changes in static pressure readings over time, which may limit the efficacy of pressure monitoring. Lastly accesses in the control group were older than those in the monitoring group (851.7 days versus 542.8 days, *P* < 0.05). This study did not answer whether more frequent monitoring is needed to see beneficial results.

Moist et al. [58] conducted a randomized controlled trial that studied 112 prevalent patients with AV graft comparing monthly Qa plus standard surveillance (dynamic venous pressure and physical examination) to standard surveillance alone. Patients were referred for intervention if flow was <650 mL/min or 20% decrease in flow in the treatment group. This study showed no difference in time to graft loss (*P* = 0.890). In multivariate analysis, only aspirin therapy was associated with an 84% reduction in risk of graft thrombosis (odds ratio 0.14; *P* = 0.002).

The randomized trial published by Ram et al. [62] in 2002 followed 101 patients with AV grafts for up to 24 months. The study population was randomized in three groups: control group, flow (Qa), or stenosis groups. All patients had monthly flow measurement with ultrasound dilution and quarterly percent stenosis by duplex ultrasound. Criteria for referral and preemptive percutaneous transluminal angioplasty (PTA) of >50% stenosis were clinical monitoring for control group, flow <600 mL/min or clinical criteria for flow group, and stenosis >50% or clinical criteria for stenosis group. Flow and stenosis groups had higher preemptive PTA rate (0.34/patient year and 0.65/patient year resp.) compared to the control group (0.22/patient year). The higher PTA rate in the intervention group failed to prolong graft survival (62% in control, 60% in flow, and 64% in stenosis group, *P* = 0.89). There was reduced rate of graft thrombosis seen in the stenosis group (47% in control, 53% flow, and 29% in stenosis group, *P* = 0.10), but it did not reach statistical significance which could have resulted from the small sample size in each group.

Malik et al. [56] conducted a multicenter randomized prospective study to observe the effect of surveillance by classic Doppler ultrasound versus clinical monitoring on patency of AVG. The sample size was 192, mean followup 392 ± 430 days. This study showed longer graft patency by regular Doppler ultrasound screening by early detection of access stenosis and intervention. But the intervention rate was quite high, therefore increasing the cost of care. An overall cost analysis was not performed.

In AVG studies, the surveillance programs have led to increased detection of stenosis and higher angioplasty rates. AVFs are known to have less frequent stenotic rates which may raise the question if surveillance programs lead to increased detection of the stenosis among fistulae. Polkinghorne et al. [59] reported a randomized, double-blind prospective controlled study to evaluate if access flow surveillance of AVF results in increased detection of AVF stenosis. Of a total of 137 patients, 68 patients were assigned to access flow measurements and 67 patients to the control group. The primary end point was angiographically significant stenosis. Access flow was measured by ultrasound dilution technique (Transonic Inc, USA). The results showed that patients in surveillance group were twice as likely to be detected with an angiographically significant stenosis compared to the controls group (control hazard ratio (HR) confidence interval (CI) (2.27, 95% 0.85–5.98, *P* = 0.09). There was a trend towards earlier detection of stenosis in the surveillance group. When using access flow alone, there was a moderate prediction of (>50%) AVF stenosis (0.78, 95% CI 0.63–0.94, *P* < 0.006). Surveillance does add to earlier recognition of a dysfunctional fistula although how this will translate into hard clinical end points is yet to be determined. This study also highlights that, although there can be difficulty in performing blinded randomized controlled trials in the care of the fistula, it is not impossible. 

Robbin et al. [60] studied 126 hemodialysis grafts in prospective randomized clinical trials comparing ultrasound surveillance and clinical monitoring in graft outcomes. 61 were randomized to receive routine clinical monitoring, and 65 were randomized to receive duplex ultrasound surveillance every four months in addition to routine clinical monitoring. The mean followup was about 22 months (21.9 months in ultrasound group and 22.9 months in control group). The ultrasound group had more frequent angioplasty (64% higher) than the control group without any added benefit in terms of graft thrombosis or surgical intervention. The hazard ratio for graft survival in the ultrasound group was 0.93 (95% CI 0.53 to 1.64). A subgroup analysis restricted to patients with virgin grafts revealed no significant difference with respect to time to graft failure (*P* = 0.32) or thrombosis-free survival (*P* = 0.72). One of the major limitations of the study was surveillance frequency which was done every four months; whether more frequent surveillance would improve graft longevity is yet to be determined. Also the spontaneous variation in flow within the access was not assessed. Without such, many accesses may have been prematurely acted upon because of the presence of a lesion which was not hemodynamically significant. Finally, the quality of monitoring which was used in both groups may have been sufficient to detect most stenosis. As stated previously, physical examination of the access by an experienced individual has high sensitivity and specificity [8–10]. Unfortunately such high-skill level is missing in most dialysis centers. See Table 2. 

The first randomized control trial that was conducted by Lumsden et al. [37] in 1997 investigated the effect of prophylactic percutaneous transluminal angioplasty (PTA) to prolong the patency of AVG in high-risk predominantly inner-city African-American dialysis patients; almost a third of the population were also diabetic. The grafts studied were not all virgin; the majority had surgical or percutaneous intervention prior to enrolment. The sample size was 64 in 2 dialysis units. Color flow duplex ultrasound was used to detect >50% stenosis, which was subsequently confirmed by angiography. Those who had >50% stenosis were randomized to balloon angioplasty versus observation; follow-up period was 12 months. There was no significant difference in patency in two groups at 6 months and 12 months. Although the demographically study populations were matched, there were more prior interventions and central stenosis in the intervention group than in control group, which may influence the result. Subgroup analysis of the 21 virgin grafts by the same group showed improved long-term patency with surveillance [68]. 

In 1999, Martin et al. [64] conducted a subset analysis of the above study. In the study population 21 patients had virgin grafts that had never undergone surgery, PTA, or thrombolysis. Among the virgin grafts, eight patients were randomized to the treatment group and 13 to the control group. The virgin groups were well matched as to age, sex, and risk factors. Stenosis of more than 50% were treated with PTA 27 times (average, 3.4 per patient) in the virgin treatment group. This study showed positive result with PTA in the virgin graft, graft patency was significantly increased (*P* > 0.0001), and the graft thrombosis significantly decreased (*P* = 0.0151) in the eight-patient virgin subset when compared with the 24-patient nonvirgin subset of the treatment group. There was a trend towards prolonged graft patency (*P* = 0.0349) and a reduction of thromboses, 0.10 versus 0.44 thromboses per patient-dialysis year, in the virgin-treatment group compared to the virgin-control group. This study has a major limitation due to very small sample size.

In a more recent study by Dember et al. [63] in 2004, 64 high-risk patients with AVG with elevated static venous pressure (≥0.4) detected by monthly measurement of static venous pressure/systolic BP ratio (SVPR) were randomized to observation and intervention groups. The intervention group received angiography and repair of the identified stenosis, whereas the observation group had stenosis repair in the event of thrombosis or clinical evidence of access dysfunction. The grafts enrolled in the study were both virgin and nonvirgin grafts with a mean age of 321 days in the intervention group and 350 in the observation group and around one-third had previous intervention across both groups. The follow-up period was 3.5 years. Although the proportion of patients with a thrombotic event was greater in the observation group (72%) than in the intervention group (44%) (*P* = 0.04), time to access abandonment did not differ significantly between the groups (hazard ratio 1.75, 95% CI 0.80–3.82, *P* = 0.16). One of the interesting findings was that access loss from infection was higher in the intervention group than in the observation group. This was noted only in nonvirgin grafts. Most of the infections occurred weeks or months after the procedure excluding the idea of direct bacterial contamination but raises the possibility that angioplasty may predispose to graft infection in the setting of occult bacteremia.

The studies on AVF and AVG have different study end points, and the major limitation has been identifying a hard end point for the interventions performed on dysfunctional access. There has been growing perception that, with increased emphasis on fistula use, the prevalence of catheter use is on the rise. In a study by Scaffaro [66], one of the end points was increased need for central venous catheters when an access fails. This does bring a new end point to the interventions being introduced for dysfunctional access. In this study, 108 patients were randomized to control and intervention groups. The control group received clinical and hemodynamic monitoring on a weekly basis; on detection of dysfunction, patient was referred to a vascular surgeon. In the intervention arm, the patients received, along with clinical and hemodynamic monitoring, a quarterly color flow duplex ultrasound study for access flow followed by angiography when access flow was under 500 mL/min. 58 patients were randomized to the control group and 53 to the intervention group. The end points were the thrombosis of the fistula and need for central venous catheters. The outcomes were evaluated at the end of 11 months. There was significant reduction in the need for central venous catheters (CVCs) in the interventional group (25.9% versus 7.5% for control and interventional group *P* = 0.021). Though there was no significant difference in the thrombosis (24.1% versus 17.0%; *P* = 0.487), the composite end point of AVF thrombosis or CVC need was reduced by the interventional strategy (44.8% versus 20.8%; *P* = 0.033). Considering that the fistula thrombosis rate is lower compared to the AVG, a followup of 11 months may have been shorter and the results may have been different with a longer followup. Since the cost of CVC placement is seldom considered in cost analyses, this study emphasizes the need for a global vascular access economic analysis.

The prospective trials involving arteriovenous fistulae are fewer compared to the AV grafts. Among the few which have been performed, Tessitore et al. [39] conducted probably the first prospective controlled open trial in 2003 to evaluate the effect of prophylactic PTA of stenosis with no known access dysfunction on survival of native virgin forearm radiocephalic AVF. Sixty-two functioning fistulas with stenosis were randomized to intervention versus controlled groups (32 versus 30, resp.). The end points were either fistula thrombosis or surgical revision due to dysfunction, but it is not clear if repeat angioplasty for access dysfunction was an end point or if not how many of the accesses had repeat angioplasty. The result showed fourfold increase in median survival and a 2.87-fold decrease in risk of failure. PTA was also associated with a significant decrease risk of hospitalization, central venous catheterization, and thrombectomy. Subsequently the same group conducted a 5-year randomized controlled trial [65] on 79 mature forearm AVF to evaluate the effect of blood flow surveillance and preemptive repair of stenosis on fistula longevity. Surveillance program included ultrasound dilution measurement of access flow on a quarterly basis, ability to maintain the prescribed blood flow rate, and urea-based access recirculation. Forty-three patients were allocated to preemptive angioplasty and 36 to the control group. Primary patency rate was improved in the intervention group (RR 3.35 with 95% CI 1.44–7.78, *P* = 0.003) and a trend towards improved secondary patency rate (RR 2.66 with 95% CI 0.98–6.85, *P* = 0.055). The study analysis also identified that higher baseline access flow (Qa) as well as higher postintervention Qa are major determinants of longer failure free interval and AVF useful life. The results suggest that the quality of the intervention is a major factor in improving patency duration.

All the studies conducted so far have small sample size, much lower than what is required to see a significant difference, and the quality of the studies reported was moderate to poor. In 2008 Tonelli et al. [69] conducted a meta-analysis of the 12 RCTs, 8 involving AVG and four trials on AVF. In fistula trials, access blood flow or ultrasound-based screening significantly decreased the access thrombosis (RR 0.47, 95% CI 0.28–0.77; 360 participants; *I* ^2^ = 8%) but not the risk of fistula loss (RR 0.65; 95% CI 0.28–1.51; *I* ^2^ = 0%) or resource use. In case of grafts there was no decrease in risk of thrombosis (RR 0.94; 95% CI 0.77–1.16; 446 participants; *I* ^2^ = 0%) or access loss (RR 1.08; 95% 0.83–1.40; *I* ^2^ = 0%). In the same year, another meta-analysis conducted by Casey et al. [70] echoed similar results.

## 4. Conclusions

A lasting and properly functioning access is crucial to provide adequate dialysis to improve the quality of life of maintenance hemodialysis patients and to reduce the huge access-related cost in this population. We are still in dilemma as to the conflicting results of observational studies and randomized control trials (RCTs) on access surveillance. It should be noted that, in all of the studies described above, the sample size used was small and much smaller than that which would have been derived using a Pearson's events-driven model which increases the sample size 4–6-fold. Sample size of around 500 is needed even for the most simplistic RCT design to see a meaningful difference with adequate power. All available RCTs have sample size less than 200 subjects, and some were as small as 30–50 allocated to one of 2-3 groups. This could be a major reason for failure to show any beneficial effect. Another major limitation could be the lack of standardized tools to assess the success of the intervention of the stenotic lesions in most of the studies. Anatomical success does not translate to improvement of the functional/physiological parameters due to elastic recoil and other factors.

The bigger question is what we are trying to achieve by performing a surveillance program? What are the hard end points? Is angioplasty the right treatment of a dysfunctional fistula? Should we consider prevention of thrombosis without improved longevity a worthy outcome? In spite of all the recent advances and increased procedures, why has the evidence for increased life of a vascular access been eluding us? All these questions lead us to the need of the hour, that is, larger multicenter scientifically sound controlled studies with adequate sample size.

## Figures and Tables

**Table 1:** Randomized trials comparing surveillance and intervention versus usual clinical monitoring and intervention.

Name	Total no. of patients	Control	Study patients	Surveillance methods tested	Primary outcome	Result
Mayer et al., 1993 [57]	70	35	35	Ultrasound evaluation of stenosis	Graft survival	Positive
Sands et al., 1999 [49]	103	41	62	Access flow, static venous pressure	Access thrombosis	Positive
Moist et al., 2003 [58]	112	53	59	Access flow, dynamic venous pressure	Access thrombosis, loss	Negative
Ram et al., 2003 [62]	101	34	67	Access flow, stenosis	Access thrombosis, survival	Negative
Roca-Tey et al., 2004 [61]*	159	65	94	Access flow	Access thrombosis	Positive
Malik et al., 2005 [56]	192	92	97	Ultrasound evaluation of stenosis	Cumulative patency	Positive
Plantinga et al., 2006 [53]*	363	185	178	Multiple	Multiple outcomes	Positive
Polkinghorne et al., 2006 [59]	137	67	68	Access flow	>50% stenosis	Negative
Robbin et al., 2006 [60]	126	61	65	Ultrasound evaluation of stenosis	Graft survival	Negative

*Prospective nonrandomized studies.

**Table 2:** Randomized trials with abnormal surveillance results and comparing intervention versus observation.

Name	Total no. of patients	Intervention	Conservative	Surveillance methods used	Primary outcome	Result
Lumsden et al., 1997 [37]	64	32	32	Color flow duplex scan	Cumulative patency	Negative
Martin et al., 1999 [64]	21	8	13	Color flow duplex scan	Virgin graft patency	Positive
Dember et al., 2004 [63]	64	32	32	Static venous pressure/systolic blood pressure ratio	Access survival	Negative
Tessitore et al., 2004 [65]	79	43	36	Access flow	Access survival, thrombosis	Positive
Scaffaro et al., 2009 [66]	108	53	58	Duplex scan	Thrombosis	Negative
